# Autoimmune Thyroid Disease and Pregnancy: The Interaction Between Genetics, Epigenetics and Environmental Factors

**DOI:** 10.3390/jcm14010190

**Published:** 2024-12-31

**Authors:** Tatjana Bogović Crnčić, Božena Ćurko-Cofek, Lara Batičić, Neva Girotto, Maja Ilić Tomaš, Antea Kršek, Ines Krištofić, Tea Štimac, Ivona Perić, Vlatka Sotošek, Sanja Klobučar

**Affiliations:** 1 Department of Nuclear Medicine, Faculty of Medicine, University of Rijeka, Braće Branchetta 20, 51000 Rijeka, Croatia; tatjanabc@medri.uniri.hr (T.B.C.); maja.ilic.tomas@uniri.hr (M.I.T.); 2 Department of Physiology, Immunology and Pathophysiology, Faculty of Medicine, University of Rijeka, Braće Branchetta 20, 51000 Rijeka, Croatia; bozena.curko.cofek@uniri.hr; 3 Department of Medical Chemistry, Biochemistry and Clinical Chemistry, Faculty of Medicine, University of Rijeka, Braće Branchetta 20, 51000 Rijeka, Croatia; 4 Department of Radiology Diagnostics, Faculty of Health Studies, University of Rijeka, Viktora Cara Emina 2, 51000 Rijeka, Croatia; neva.girotto@uniri.hr; 5 Faculty of Medicine, University of Rijeka, Braće Branchetta 20, 51000 Rijeka, Croatia; antea.krsek@uniri.hr; 6 Department of Obstetrics and Gynecology, Faculty of Medicine, University of Rijeka, Braće Branchetta 20, 51000 Rijeka, Croatia; ines.kristofic@uniri.hr (I.K.); tea.stimac@uniri.hr (T.Š.); 7 Department of Endocrinology, Diabetes and Metabolic Diseases, Clinical Hospital Centre Rijeka, 51000 Rijeka, Croatia; ivona.peric@uniri.hr (I.P.); sanja.klobucar@uniri.hr (S.K.); 8 Department of Anesthesiology, Reanimatology, Emergency and Intensive Care Medicine, University of Rijeka, Braće Branchetta 20, 51000 Rijeka, Croatia; 9 Department of Clinical Medical Sciences II, Faculty of Health Studies, University of Rijeka, Viktora Cara Emina 2, 51000 Rijeka, Croatia; 10 Department of Internal Medicine, Faculty of Medicine, University of Rijeka, Braće Branchetta 20, 51000 Rijeka, Croatia

**Keywords:** autoimmune thyroid disease, Graves’ disease, Hashimoto’s thyroiditis, maternal-fetal health, pregnancy, thyroid dysfunction

## Abstract

Autoimmune thyroid disease (AITD) is the leading cause of thyroid dysfunction globally, characterized primarily by two distinct clinical manifestations: Hashimoto’s thyroiditis (HT) and Graves’ disease (GD). The prevalence of AITD is approximately twice as high in women compared to men, with a particularly pronounced risk during the reproductive years. Pregnancy exerts profound effects on thyroid physiology and immune regulation due to hormonal fluctuations and immune adaptations aimed at fostering maternal–fetal tolerance, potentially triggering or exacerbating AITD. The impact of AITD on pregnancy outcomes is multifaceted. Both HT and GD have been associated with adverse obstetric and neonatal outcomes, including miscarriage, preterm delivery, preeclampsia and fetal growth restriction. Inadequately managed AITD can also affect fetal neurodevelopment due to disrupted maternal thyroid hormone availability during critical periods of brain maturation. This review explores the complex interplay between the genetic, epigenetic and environmental factors that drive AITD during pregnancy, highlighting their roles in disease development and impacts on pregnancy outcomes. Gaining a deeper understanding of these mechanisms is crucial for improving diagnostic tools, treatment options and preventive measures to enhance the health and well-being of both the mother and the newborn.

## 1. Introduction

Autoimmune thyroid disease (AITD) is caused by dysregulation of the immune system which results in an autoimmune attack on the thyroid gland. Hypothyroidism and hyperthyroidism are the two clinical faces of AITD, the most common cause of thyroid dysfunction involving various cellular and humoral interactions within thyroid tissue, mainly categorized as Hashimoto’s thyroiditis (HT) and Graves’ disease (GD). The global prevalence of AITD has increased and now affects approximately 5% of the world’s population. However, despite having a history more than a century long, the pathogenesis of both entities is still not fully understood [1].

The development of AITD is believed to result from a complex interplay of genetic predisposition and environmental factors that lead to a breakdown in immune tolerance and trigger an autoimmune response against the thyroid gland. Central to this process is the disruption of tolerance to self-antigens involving a complex network of interactions between thyroid follicular cells (TFCs), stromal cells and immune cells [2].

In patients with AITD, cellular and humoral immunity play a role. As a result, lymphocytes infiltrate the thyroid parenchyma, targeting thyroid antigens such as the sodium iodide symporter, thyroglobulin (Tg), thyroid-stimulating hormone receptor (TSH-R) and the enzyme thyroid peroxidase (TPO) [3,4]. One of the components that have an immunomodulatory effect is thyroid hormone status, as immune cells contain receptors for thyroid hormones [5]. In AITD, other factors such as genetic susceptibility through the polymorphism of human leukocyte antigen (HLA) system genes and thyroid-specific AITD susceptibility genes also appear to play a role, together with epigenetic and environmental factors including excessive iodine consumption, selenium [6,7] and vitamin D deficiency [8], infectious agents [9] and gut microbiota dysbiosis [10,11]. A link between HT and interferon (IFN)-α, lithium or amiodarone therapy has also been recognized [12,13], and even a possible connection with climate factors, with an increased incidence in colder regions of the world, has been suggested [14]. Smoking and stress are also associated with the risk of GD [15,16]. Thyroid hormones play an important role in both innate immunity and the adaptive immune response. Both disorders, GD and HT, are defined by circulating thyroid-specific antibodies and the infiltration of autoreactive lymphocytes into the thyroid gland and are of great concern during pregnancy as they can greatly affect both maternal and fetal outcomes [2,9,15]. The involvement of environmental factors in the development and expression of AITD is important in terms of disease progression and pregnancy outcome. Therefore, lifestyle intervention with the above modifiable risk factors could help to maintain low AITD risk and improve pregnancy outcomes.

The prevalence of AITD in the general population is estimated to be approximately 5–10%, with variations depending on demographic and geographic factors. AITD is a common endocrine disorder among pregnant women, with a prevalence ranging from 2% to 5% [1,2,3,4]. Its clinical significance during pregnancy is substantial, as thyroid dysfunction can impact both maternal and fetal health. For instance, maternal hypothyroidism is associated with adverse pregnancy outcomes, including preterm birth, low birth weight and impaired neurodevelopment in the newborn [3,5]. On the other hand, untreated maternal hyperthyroidism induces risks such as preeclampsia, preterm delivery and fetal growth restriction [4,5,14]. Therefore, the early identification and management of AITD in pregnancy are critical to optimizing outcomes for both the mother and the child. The aim of this review is to describe the main genetic, epigenetic and environmental factors involved in the pathogenesis of AITD, focusing on their role in pregnancy in patients with thyroid autoimmunity.

## 2. Clinical Forms of Autoimmune Thyroid Diseases

The clinical presentations of AITD are diverse and can manifest as hyperthyroidism (e.g., GD), hypothyroidism (e.g., HT) or a combination of thyrotoxicosis and hypothyroidism, as seen in postpartum thyroiditis (PPT). Importantly, while GD is commonly associated with hyperthyroidism, it can also present with hypothyroidism in cases involving blocking thyroid receptor antibodies (TRAb). Furthermore, in GD, phases of hyperthyroidism and hypothyroidism may alternate or overlap, which is also a common feature of this condition.

### 2.1. Autoimmune Hyperthyroidism

GD was named after Robert Graves, an Irish physician, who first described this form of hyperthyroidism in 1835. There are differences in incidence between genders (women 5–15% and men 1–5%) [17], especially during the reproductive period [18]. GD includes a range of symptoms including hyperthyroidism, diffuse goiter, Graves’ orbitopathy (GO) and dermopathy [19,20] and is the most common cause of hyperthyroidism, representing 60% to 80% of cases of hyperthyroidism [21]. It is most common between the ages of 40 and 60 [22]. GD is characterized by a hypoechoic and inhomogeneous parenchyma, often accompanied by thyroid gland enlargement, as seen on ultrasound. A family history of thyroid disease, especially in maternal relatives, is associated with an increased risk of GD [23]. GD occurs due to the presence of stimulatory anti-TSH-R autoantibodies (TRAbs), which bind to and activate the TSH-R. Activation of the receptor leads to cell hyperplasia and hypertrophy of the thyroid follicles and, consequently, to the increased synthesis of thyroid hormones. TRAbs are mainly synthesized by B lymphocytes in the thyroid gland but can also be produced in lymph nodes and bone marrow. After the sensitization of T lymphocytes with thyroid antigens, B lymphocytes are activated [23].

The pathology of thyroid diseases lies in the interaction between thyroid hormones, the immune system and minerals. The minerals that are mostly involved in this interaction are magnesium, selenium, calcium, zinc, iron and copper [24]. Zinc participates in the immune system as an immunomodulator, and its deficiency suppresses the innate and adaptive response [24]. In hyperthyroidism, the impaired mitochondrial function in hyperthyroidism is likely related to deficiencies in magnesium, selenium and the antioxidant coenzyme Q10. [24]. Clinically, hyperthyroidism manifests itself through general signs of metabolic acceleration: nervousness, hyperactivity, mood swings, sleep disorders, sensitivity to heat, muscle weakness and diarrhea.

### 2.2. Autoimmune Hypothyroidism

In his report, from 1912, Hakaru Hashimoto was the first to describe the lymphocytic infiltration of the enlarged thyroid gland and, thus, introduced the term lymphomatous goiter [25]. It is now considered the most common autoimmune disease characterized by the cellular immune response and lymphocytic infiltration resulting in the gradual destruction of thyroid tissue, and often leading to hypothyroidism [26,27].

The incidence of HT is estimated at 0.3–1.5 cases per 1000 people [28], with a higher prevalence in women (5–15% vs. 1–5% in men), especially those middle aged and living in iodine-sufficient areas [29]. Recent data also suggest a higher incidence in the same geographical areas compared to prior studies, and an overall increase in the incidence of AITD in recent years [30].

Typical ultrasonography findings include enlargement of the thyroid gland with hypoechoic and inhomogeneous parenchyma [31]. The risk of HT is higher in relatives, especially those with other autoimmune diseases. Although thyroid autoantibody titers do not seem to play a significant role in the pathogenesis of this disease, thyroid peroxidase antibodies (TPOAbs) are present in almost 90% of cases and 5–20% of women of reproductive age are considered positive for thyroid autoimmunity [32].

Subclinical cases of HT are defined by elevated thyroid-stimulating hormone (TSH) and normal thyroid hormone levels, without typical symptoms, but with an increased rate of cardiovascular morbidity [33]. It is estimated that hypothyroidism develops in approximately 20–30% of patients with HT. Interestingly, some large population studies suggest that subclinical hypothyroidism in the elderly population may be associated with reduced morbidity [34].

When it occurs clinically, hypothyroidism is manifested by general signs of a metabolic slowdown (weight gain, slowed heart rate and bowel movements, edema, fatigue, normocytic anemia, skin changes with hair and body hair loss and anovulatory cycles with menorrhagia) and memory impairment [35]. In an early stage, symptoms and signs of thyrotoxicosis, due to a massive release of thyroid hormones from damaged thyroid cells, may also occur [36].

The diagnosis of HT is based on clinical symptoms, the presence of circulating antibodies to thyroid antigens (mainly TPOAbs and TgAbs) and / or a typical sonographic appearance of the thyroid gland. Ultrasonography is particularly important in establishing the diagnosis in patients with seronegative HT, which occurs in 5–10% of cases [28].

## 3. Pregnancy and Autoimmune Thyroid Disease

A healthy pregnancy and proper fetal development are of the utmost importance. During pregnancy, maintaining optimal thyroid function is crucial for the health of the mother and the development of the fetus. Pregnancy places increased demands on the thyroid gland as thyroid hormones play a critical role in fetal growth and neurological development, especially in the first trimester when the fetus is completely dependent on maternal thyroid hormones. Thyroid hormones are critical for fetal brain development, and the disruption of thyroid function due to AITD can have detrimental effects on the child’s neurological outcomes [37].

The course and progression of AITD can vary during pregnancy and may change after delivery. This is because the mother’s immune system undergoes significant changes during pregnancy, with metabolic adaptations that help maintain immune tolerance to the fetus, which carries paternal antigens on its cells [38]. Therefore, despite transient immunosuppression, maternal immunity must be able to maintain effective protection of both the mother and the fetus against infection while protecting the fetus from the maternal immune system. It is, therefore, assumed that cellular immunity is reduced during pregnancy in order not to reject the fetus. However, these changes in the immune status of the pregnant woman appear to influence the course of the autoimmune disease itself [39]. The natural history of GD includes improvement in the second half of the pregnancy and worsening or recurrence after delivery, which is partly explained by the immunomodulation of the maternal immune response during pregnancy [40]. However, the pathophysiology and mechanisms underlying these changes are unknown and require further research.

### 3.1. Autoimmune Hyperthyroidism in Pregnancy

Hyperthyroidism during pregnancy is rare, occurring in 1–3/1000 pregnancies (its prevalence is 0.1–0.3%), depending on whether overt or subclinical forms are considered [41,42]. The most common cause is GD, which is estimated to account for 85–95% of clinically significant cases of hyperthyroidism [43]. Since some authors have shown that the functional activity of TRAbs changes from stimulation to inhibition [44,45,46], this means that GD should be well monitored during pregnancy. The symptoms of GD in pregnancy are no different from those in non-pregnant women but can be confused with the symptoms of pregnancy [47].

**Figure 1:**
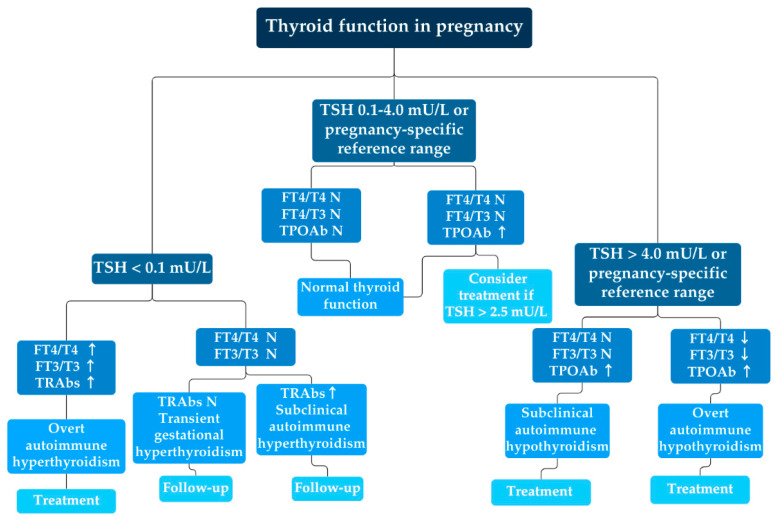
Assessment of thyroid function during pregnancy. The figure shows a schematic representation of thyroid function tests during pregnancy according to the American Thyroid Association (2017) [58]. Overt autoimmune hyperthyroidism is characterized by a thyroid stimulating hormone (TSH) level of less than 0.1 mU/L and elevated thyroid hormones and thyroid receptor antibodies (TRAbs), indicating the need for treatment. Transient gestational thyrotoxicosis is suspected when normal thyroid hormone levels are observed while TSH is suppressed and TRAb levels are in the reference range. If TRAb levels are elevated, subclinical autoimmune hyperthyroidism should be considered. In both cases, a follow-up examination is required. (Abbreviations: TSH—thyroid stimulating hormone, T4—thyroxine, FT4—free thyroxine, T3—triiodothyronine, FT3—free triiodothyronine, TRAbs—thyroid receptor antibodies, TPOAb—thyroid peroxidase antibody, and N—normal, ↑—elevated, ↓—decreased).

Since maternal thyroid function undergoes significant changes during pregnancy, different approaches are needed when interpreting thyroid function tests in pregnant women compared to non-pregnant women [47]. The evaluation begins with the TSH level, which is often lowered during pregnancy due to human chorionic gonadotropin (hCG) stimulation. TSH levels are trimester-specific, and the disease is characterized by an elevated thyroxine (T4) and triiodothyronine (T3) level and suppressed serum TSH [48]. Free T4 (FT4) and free T3 (FT3) levels are evaluated, and total T4, T3 and thyroxin-binding globulin (TBG) levels may be clinically useful when available [41,43,49,50,51,52,53]. In GD, (stimulating) TRAbs are usually measurable and can be used to confirm the diagnosis and differentiate it from transient gestational thyrotoxicosis [50] (Figure 1). Uncontrolled GD during pregnancy can lead to significant maternal, fetal and neonatal complications [49].

Since the fetal thyroid gland is mature from 20 weeks onwards, it could respond to the influence of both antithyroid drugs (ATDs) and TRAbs [47]. Fetal hyperthyroidism could be caused by the transplacental passage of excess thyroid hormone or by the activation of the thyroid gland with stimulating TRAbs [54,55,56]. Autoimmune hyperthyroidism has even been documented in babies born to mothers who were treated for GD several years earlier but who still had detectable circulating thyroid receptor antibodies [57]. Serious complications are usually due to fetal hyperthyroidism, which can even lead to fetal death [58]. Obstetric ultrasound offers the possibility of screening for thyroid dysfunction. The findings may include an enlarged thyroid gland, intrauterine growth restriction, hydrops, advanced bone maturity, heart failure, goiter and oligohydramnios [43,51,53,54]. In addition, thyroid hormones have an impact on neurodevelopmental abnormalities by regulating the migration, growth and differentiation of fetal neurons [54]. The guidelines of the American Thyroid Association (2017) and the European Thyroid Association (2018) recommend monitoring TRAb levels in maternal blood during pregnancy and the additional testing of fetal thyroid function in newborns shortly after birth [58,59,60].

Significant thyroid enlargement can lead to difficulties in fetal head mobility and, possibly, abnormal presentation at birth, dysphagia leading to polyhydramnios and, consequently, premature birth, or the compression of immature tracheal cartilage leading to airway obstruction. Polyhydramnios can trigger and promote premature birth, the leading cause of neonatal morbidity and mortality [47].

Neonatal hyperthyroidism because of the persistence of maternal TRAb (half-life about 2 weeks) can occur in 1–5% of infants. After the disappearance of maternal TRAbs, neonatal central hypothyroidism may occur due to the persistent suppression of fetal pituitary TSH production [41].

Maternal complications of hyperthyroidism include hypertension, preeclampsia and placental abruption [50]. Two other serious complications are thyroid storm, which is characterized by altered mental status, hyperthermia, tachycardia, left dentate dysfunction, multiorgan failure and congestive heart failure [41], which can be diagnosed in 10% of untreated severe hyperthyroidism cases due to the increased cardiac workload. The much higher rate in pregnant women compared with non-pregnant women could be explained by co-existing pregnancy complications (severe preeclampsia, anemia, hemorrhage, etc.) [61].

Hyperthyroidism in pregnancy is treated with drugs that inhibit the excessive synthesis of thyroid hormones [50]. All ATDs cross the placenta and can, therefore, affect the fetal thyroid gland, so the lowest dose should be used to maintain FT4 levels [62]. Treatment with ATDs is necessary despite the potential teratogenicity due to the negative impact on maternal health and the risk of fetal loss in untreated overt hyperthyroidism [48,52]. Hypothyroidism caused by ATDs is usually transient and corrects itself when the drug is metabolized after birth, although this requires close monitoring [58].

Due to the immunosuppressive effect of pregnancy, GD is in remission in many women towards the end of pregnancy [52]. As with other autoimmune diseases, GD typically improves due to immune tolerance during pregnancy, which aims to prevent the fetus from being rejected as a foreign body by immunologic molecules of trophoblastic origin and T-cell subsets [T-regulatory cells (T-reg)] that arise in the decidua [41,42,63]. T-reg cells induce transient immunosuppression in the maternal circulation, which can attenuate the onset of GD [41]. After delivery, the abrupt drop in T-reg cells is the reason for the postpartum resurgence of autoimmunity and the exacerbation of GD [41]. In some cases, TRAbs may have an inhibitory effect instead of stimulating the thyroid gland. After delivery, there is a risk of exacerbation or relapse due to the rebound of the maternal immune system, usually 7–9 months after pregnancy [64].

Maternal overt or subclinical hypothyroidism is defined when the TSH level is above the population-based pregnancy-specific reference range; if this is not available, a TSH cut-off value of 4.0 mU/L is recommended. A TSH value between 0.1 and 4.0 mU/L or a population-based pregnancy-specific reference range with normal thyroid hormone and TPOAb indicates normal thyroid function and requires no further testing. However, if the TSH level is above 2.5 mU/L and the TPOAbs are elevated, levothyroxine therapy could be considered. On the other hand, if TSH is above 4.0 mU/L or the population-based pregnancy-specific reference range and TPOAbs are elevated, subclinical (normal thyroid hormone levels) or overt (reduced thyroid hormone levels) autoimmune hypothyroidism is diagnosed and supplementation with levothyroxine is recommended. 

### 3.2. Autoimmune Hypothyroidism in Pregnancy

It is estimated that 5–15% of healthy women of reproductive age are affected by HT [65]. In addition, approximately 2–17% of pregnant women have increased levels of TPOAb and TgAb. These antibodies have been shown to gradually decrease during pregnancy, reaching their lowest levels in the third trimester, then rising again as early as 6 weeks post-partum and returning to pre-pregnancy levels approximately 12 weeks later [66].

The inability of the thyroid gland to cope with increased hormonal demands during pregnancy often results in subclinical or overt hypothyroidism, which has significant implications for maternal and fetal health, including an increased risk of miscarriage, preterm labor, placental abruption, preeclampsia and gestational hypertension, postpartum thyroid dysfunction and other risks [67]. In developing fetuses, an association between low birth weight, prematurity, developmental delays and stillbirths with maternal hypothyroidism has been confirmed [68]. Maternal hypothyroidism can also have serious effects on the child’s cognitive development, as optimal levels of thyroid hormones are necessary to regulate certain processes in the fetal brain, such as neuronal migration and myelination [69,70]. Maternal hypothyroidism is defined as a TSH level that is above the pregnancy-specific reference range. In areas where a pregnancy-specific reference range is not available, a TSH cut-off value of 4.0 mU/L should be used (Figure 1) [58,68]. Women who are euthyroid but tested positive for TPOAb prior to pregnancy are at increased risk of developing elevated TSH during pregnancy and require regular TSH monitoring [69]. In addition, the presence of thyroid autoantibodies in women is associated with a two- to threefold increased risk of pregnancy loss, although the mechanism of action is unknown [71,72]. Even in euthyroid pregnant women, an increased risk of preterm delivery has been found in the presence of thyroid autoantibodies [73].

While overt hypothyroidism affects about 0.2–0.6% of pregnant women [74], subclinical hypothyroidism (SH) is more common and occurs in 3.5–18% of pregnancies [75]. It is characterized by normal levels of free thyroid hormones and TSH levels above the pregnancy reference range [59]. Korevaar et al., in a meta-analysis of 19 cohort studies, found an increased risk of preterm delivery in pregnant women with SH, with an odds ratio 1.04 (95%CI, 1.00–1.09) for each one standard deviation increase in TSH. The risk was also increased in the presence of TPO antibodies [76].

There is a broad consensus on the treatment of overt hypothyroidism in pregnancy. In SH, levothyroxine (LT4) treatment is indicated in TPOAb-positive pregnant women with TSH values above the pregnancy-specific reference range and in TPOAb-negative pregnant women with TSH values above 10.0 mU/L. The question of whether TPO-negative women should be treated with SH remains controversial. The studies by Zhu et al. and Magri et al. have shown that women with SH and without thyroid autoimmunity have a higher prevalence of gestational diabetes, anemia, preeclampsia and fetuses small for gestational age [77,78]. LT4 treatment may be considered in TPOAb-positive pregnant women if TSH levels are above 2.5 mU/L and in TPOAb-negative pregnant women if TSH levels are above the pregnancy-specific reference range but do not exceed 10.0 mU/L (Figure 1) [58,68]. In addition, euthyroid women who test positive for TPOAb and/or TgAb are at risk of PPT within the first year after delivery due to an immune system rebound. Pregnant women with higher TPOAb levels have a higher risk of developing PPT [59]. The diagnosis of HT during pregnancy is difficult because the symptoms of hypothyroidism are like those commonly seen in pregnant women, and the timely determination of serum hormone concentration and TPO autoantibody titer is crucial. Pregnant women with subclinical hypothyroidism or borderline TSH levels early in pregnancy may not be able to meet the increased demand for thyroid hormones and may show signs of thyroid insufficiency during pregnancy [59].

It is important to emphasize that maternal and fetal thyroid functions are closely linked. The fetal thyroid gland concentrates and begins to fully synthesize thyroid hormones only after 18–20 weeks of gestation, but even after that, the fetus requires both maternal and its own thyroid hormones for normal development [74,79]. The adverse effects are thought to depend on the severity and timing of the maternal hormone deficiency. Therefore, overt hypothyroidism should be diagnosed early in pregnancy and treated promptly [74].

Given that AITD is the most common autoimmune disorder in young women, and considering its potential consequences—along with the fact that it can remain undiagnosed (and untreated) in its subclinical form—systematic screening for AITD during pregnancy has been recommended. Several studies have demonstrated the ineffectiveness of selective screening in identifying affected individuals. However, there is still no consensus in favor of the systematic screening of thyroid function in all pregnant women [80].

The complex pathophysiology of thyroid autoimmunity and the effects on thyroid dysfunction during pregnancy are still far from clear. Therefore, further studies are needed to help us uncover the underlying mechanisms and all factors involved.

## 4. Genetic Factors and Autoimmune Thyroid Diseases

Although HT and GD manifest with contrasting clinical presentations and represent opposite ends of the clinical spectrum of AITD, they share a common complex etiology involving the reciprocal interaction of the genetic basis with epigenetic and environmental factors [81]. AITD is considered a familial disease, as the familial clustering of AITD (risk ratio between siblings) is estimated to be 5.9 to >10, with a value of >5.0 considered significant. A high recurrence rate was found in first- and second-degree relatives of patients with AITD; among GD patients, 6.1% of first-degree relatives had GD, and among HT patients, 4.9% of first-degree relatives were affected [82]. Numerous recent studies have contributed significantly to a better understanding of the role of the genetic component in the development of mechanisms that promote thyroid autoimmunity [81,82,83,84,85]. This is certainly the basis for the earlier identification of individuals at increased risk of developing AITD and for earlier intervention, but also for the development of targeted therapeutic options.

### 4.1. Immune-Related Genes

HLA complex is a highly polymorphic genetic region that encodes several proteins that play a critical role in regulating the immune response and cellular self-recognition through antigen presentation and lymphocyte activation. More than 70 diseases have been linked to HLA polymorphisms, and many autoimmune diseases are associated with specific variations of the HLA class II gene [83]. Currently, the HLA-DR3 subtype is most consistently correlated with a higher risk of developing AITD. Evidence of this is the fact that 40–50% of GD patients have the HLA-DR3 gene, as opposed to 15–30% of the general population. Although the link between HLA-DR3 and HT was initially less conclusive, it has also been demonstrated [81,86].

The CTLA-4 gene is important for immune regulation as it plays a role as a downregulator of the T-cell-mediated immune response, while CD152 is one of the expression products of the gene encoding the synthesis of CTLA-4 [82]. The mechanism by which CTLA-4 downregulates T-cell activation is by binding to the intracellular domain of CD152 during the early stage of activation and mediating the negative signaling that inhibits T-cell activation. The increased expression of CD152 on T cells in the late phase of immune activation leads to competition with CD28 for binding to B7 [85]. Consequently, even a subtle polymorphism in the CTLA-4 coding region can contribute to the development of decreased CTLA-4 expression, leading to a hyperactive and self-destructive immune response with increased production and secretion of thyroid autoantibodies and, eventually, AITD [82].

The CD40 is a transmembrane cell surface receptor that is classically expressed on B lymphocytes and other antigen-presenting cells and binds specifically to CD40 ligand (CD40L) on the surface of target cells [82]. Stimulation of the CD40 molecule controls the proliferation, expansion and activation of B cells. It also up-regulates major histocompatibility complex class II (MHC II) on B cells, drives plasma cell differentiation and promotes immunoglobulin isotype class switching and antibody secretion [81]. Some variants of the CD40 single nucleotide polymorphism (SNP) are associated with an increased risk of developing AITD. The variant rs1883832 was most clearly associated with a significant risk of GD, but no association with HT was found [82]. Studies, including immunohistochemistry and flow cytometry, confirmed the increased expression of CD40 in thyroid cells from individuals with GD (particularly in epithelial cells, follicular cells and fibroblasts). One possible mechanism that could explain the susceptibility to AITD is based on the hypothesis that autoantigens from GD patients are processed by the affected thyroid follicular epithelial cells and presented to infiltrating thyroid tissue T cells to induce their activation, while CD40/CD40L enhances the immune response during this process [81,87,88].

The PTPN22 gene plays a role in encoding lymphoid-specific tyrosine phosphatase (LYP), and polymorphism in this genetic region has been shown to have a strong and consistent association with the development of numerous autoimmune diseases. LYP is capable of suppressing kinases that mediate the activation and regulation of T lymphocytes but also plays an important role in B-lymphocyte signaling and is involved at multiple levels in the T-cell receptor signaling and activation cascade. Therefore, after HLA, the PTPN22 gene is the one most strongly associated with increased AITD risk, as it has regulatory effects on multiple cell types involved in the immune response and various signaling pathways [82].

The FOXP3 gene codes to produce the FOXP3 protein, which attaches to specific areas of DNA and helps to control the activities of genes involved in the regulation of the immune system. It is a crucial factor in the physiological development of T-regs. It has been shown that certain nucleotide polymorphism variants weaken the inhibitory function of T-regs and, thus, favor the development of an autoimmune reaction [89,90].

### 4.2. Thyroid-Specific Genes

The Tg gene is located on chromosome 8q24 and encodes a large glycoprotein homodimer molecule of Tg, which is the most quantitatively dominant autoantigen in the thyroid gland. Tg represents the matrix for thyroid hormone synthesis and the basic storage molecule for newly synthesized T3 and T4 [82]. TSH-R represents a major autoantigen of the thyroid gland, so many studies have been conducted to investigate the association between different TSH-R polymorphisms and the propensity to develop AITD, especially GD [82].

Intron 1 of the TSH-R gene was marked as a region of interest for GD and five GD-associated SNPs were mapped to TSH-R intron 1: rs179247, rs2284720, rs12101255, rs12101261 and rs2268458 [81]. In the current literature, two pathophysiological mechanisms have been proposed to explain the association between intron 1 variants and an increased risk of thyroid autoimmunity. The first mechanism is based on the disruption of peripheral tolerance and the development of an autoimmune reaction to TSH-R due to alterations in mRNA splicing of thyroid genes. The other theory is based on a lack of central immune tolerance due to a reduced expression of TSH-R in the thymus in individuals with the aforementioned SPNs [81,82].

The Tg gene contains more than 16,000 SNPs and certain Tg SNPs and allelic variations have been shown to correlate with AITD [82]. One of the possible underlying mechanisms is thought to be alternate endosomal Tg degradation leading to the release of immunogenic peptides [82]. Lee at al. note that in sequencing studies of the 5`UTR (untranslated region) of Tg, there is an A/G SNP at position 1623 (rs180195) that is strongly associated with AITD since it disrupts a regulatory element within the Tg promoter [81]. Furthermore, they mentioned three missense polymorphisms responsible for altering the amino acid sequence in the Tg molecule, possibly triggering the ER-to-lysosome-associated degradation (ERLAD) pathway of Tg and the generation of peptides that have an increasing binding affinity for HLA-DRb-Arg74 [81], thus resulting in significantly increased probability of GD occurrence [91].

The TPO gene is located on chromosome 2p25 and encodes TPO, a glycosylated hemoporotein located in the apical membrane of thyrocytes and consisting of a large extra-cellular, a short transmembrane domain and an intracellular C-terminal region. Its function is to catalyze the iodination of the tyrosine residues of Tg. Although TPO is considered one of the major thyroid antigens and AITD can result from several mechanisms (including total TPO dysfunction, heme cofactor binding disruption, inability to interact with the Tg substrate and disruption of localization with subcellular placement), the number of studies reporting the association of TPO gene polymorphisms with the development and prognosis of AITD is modest [82]. In their study, Tomari et al. genotyped eight single nucleotide polymorphisms in the TPO gene and demonstrated that TPO rs2071400 T carriers (CT + TT genotypes) and the TPO rs2071403 GG genotype were more common in individuals with AITD, including GD and HD patients [92]. However, no significant association was found between the SNPs and the prognosis of AITD. On the other hand, serum levels of TPOAb were significantly higher in AITD patients who were TPO rs2071400 T carriers (CT + TT genotypes) and TPO rs2048722 T carriers (CT + TT genotypes) than in those with the CC genotype [92].

### 4.3. Epigenetics Factors in Autoimmune Thyroid Disease

Since the AITD risk for some of the previously mentioned genes is rather low, it is thought that a synergy of environmental factors and genetic susceptibility may be necessary to trigger the development of AITD [82]. Epigenetics is considered a key factor in the integration of these genetic and environmental elements. Epigenetics provides insights into the mechanisms involved in the regulation of gene expression without changes in the underlying DNA sequence. The most important epigenetic mechanisms in AITD include DNA methylation, histone modifications, RNA interference by non-coding RNAs and inactivation of the X chromosome [93].

DNA methylation refers to the process of binding methyl groups to specific DNA regions, usually silencing gene expression [94]. In AITD, DNA methylation abnormalities affect the immune-related and thyroid-specific genes mentioned earlier. For example, in GD, the hypomethylation of genes related to immune activation contributes to the production of thyroid-stimulating antibodies. On the other hand, some genetic polymorphisms of DNA methylation-regulating genes can also lead to the dysfunction and maldevelopment of the DNA methylation process, further increasing the susceptibility to disease. Studies on DNA methylation are still limited and show high variability, but all agree with the conclusion that abnormal DNA methylation plays an important role in the pathogenesis of AITD [95].

Histone modifications play an important role in the control of chromatin compaction, nucleosome dynamics and DNA repair and can directly regulate transcription [96]. Like DNA methylation, histone modifications are highly dynamic and can alter gene expression. Recent studies have provided evidence for their role in the modulation of immune tolerance and the development of autoimmune diseases [97]. Further research is needed to clarify the role of histone modifications in the pathogenesis of AITD, but also to elucidate their potential role as diagnostic biomarkers and predictors of treatment success in AITD patients [92].

Non-coding RNAs are small RNAs, especially microRNAs with a length of 18 to 25 nucleotides, which play an important role in the post-transcriptional regulation of gene expression [98]. Some miRNAs such as miR-223-3p and miR-155-5p also play an important role in regulating immune function and maintaining immune homeostasis. There-fore, it is not surprising that the abnormal expression of miRNAs involved in immune function may contribute to the development of autoimmune diseases [99,100,101]

Several recent studies have provided evidence of the abnormal expression of miR-155-5p and miR-146a-5p in AITD patients [102,103]. The abnormal expression of miR-155-5p and miR-146a-5p can certainly promote the development of AITD by disrupting immune homeostasis and immune tolerance [92]. Bernecker et al. found that patients with GD and HT had significantly lower levels of miR-146a-5p and miR-155-5p in thyroid tissue [104]. Currently, there are many studies investigating the differentially expressed miRNAs in AITD patients, but studies specifically exploring their clinical utility should be prioritized.

Inactivation of the X chromosome (XCI) is an important epigenetic trait that occurs randomly in females and involves the transcriptional silencing of an X chromosome. AITD has been shown to affect female patients more frequently, raising the question of a possible key role of XCI in the developmental process. An underlying mechanism could be distorted XCI, which may lead to a loss of balance of gene products and immune tolerance. Several previous studies have demonstrated an increased frequency of skewed XCI in AITD patients, while Ishido at al. found a non-significant difference in prevalence between GD patients and healthy individuals but demonstrated a correlation between skewed X chromosome and GD progression [105].

### 4.4. Environmental Factors

**Figure 2:**
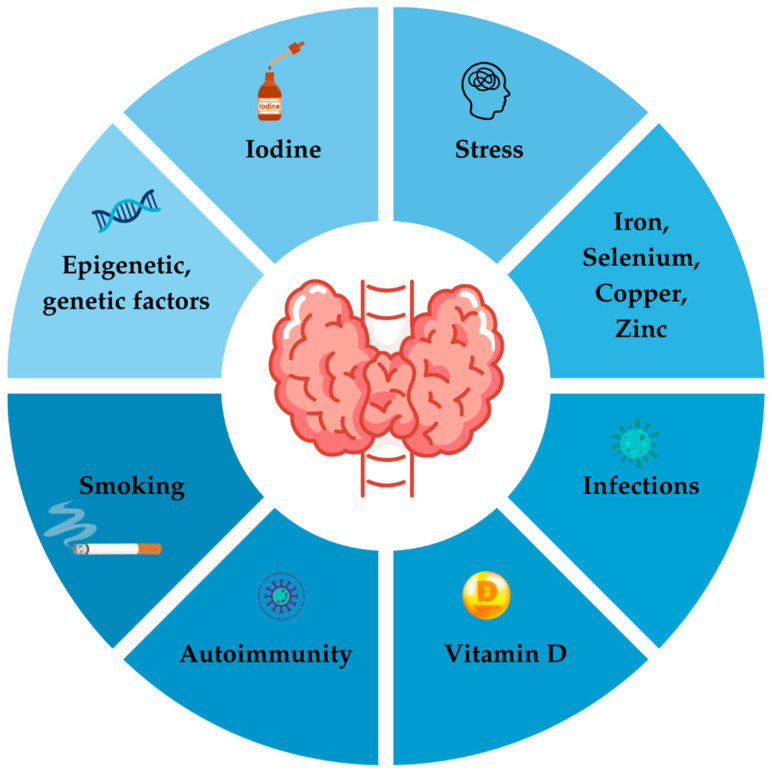
Key factors influencing thyroid function. Figure illustrates the most critical physiological, environmental, genetic and lifestyle factors that impact thyroid function.

The key environmental factors involved in development of AITD are iodine excess, deficiency of selenium, iron and vitamin D, smoking, stress and infections [106,107] as shown on Figure 2.

#### 4.4.1. Iodine Intake and Autoimmune Thyroid Disease

Of all the environmental factors that influence AITD, iodine is the most important because it is necessary for the synthesis of thyroid hormones [106,107]. Iodine is incorporated into the tyrosine residues of Tg, which ultimately leads to the production of the hormones T3 and T4. Iodine deficiency continues to be an important public issue and a common health problem worldwide. It is associated with decreased serum levels of thyroid hormones and leads to goiter, hypothyroidism, irreversible cognitive impairment, cretinism and even cancer [16,24]. On the other hand, it is also known that excess iodine can lead to thyroid dysfunction, resulting in either hypothyroidism or thyrotoxicosis through a transient destructive effect on thyroid cells or even hyperthyroidism if autonomously functioning thyroid tissue is present [108]. In addition, evidence has accumulated over the years that iodine prophylaxis is associated with an increased prevalence of thyroid autoimmunity, especially in areas previously deficient in iodine [109,110]. The exact cause of thyroid autoimmunity in relation to iodine is still not fully understood. The thyroid autoimmune process may be exacerbated or triggered by excessive or insufficient iodine intake [111]. The increasing prevalence and variety of thyroid disorders suggest possible negative effects of increased iodine intake. This trend is consistent with observations from studies conducted in both the United States and Europe [112,113]. There are data suggesting that iodine may be involved in triggering the process of lymphocytic intrathyroidal infiltration. One of the possible explanations is that iodine could increase the production of cytokines such as tumor necrosis factor (TNF)-alpha and chemokines and cause cell damage through oxidative stress [24,114,115]. In addition, a more-than-adequate iodine intake in the mouse model increases the expression of the intercellular adhesion molecule 1 on the thyroid cell, which leads to the increased infiltration of mononuclear cells and inflammation [116]. It has also been suggested that iodine may induce Th17 T cells in the thyroid gland and impair the development of T-reg cells and that, in excessive amounts, it could increase the antigenicity of thyroglobulin by altering its conformation [117]. In addition, increased TPOAb and TgAb titers have been found in regions with excessive iodine intake, also suggesting that excessive iodine consumption could lead to the development of AITD [110,118]. In Denmark, the iodized salt program in regions with moderate and mild iodine deficiency led to a steady increase in the incidence of hypothyroidism in young and middle-aged subjects [119]. On the other hand, there are studies that have found no change in thyroid autoimmunity over time following salt iodization [120,121]. A national cross-sectional survey in China in 2020 showed that after two decades of the salt iodization program, the prevalence of positive TPOAb was low and a more-than-adequate iodine intake was inversely related to TPOAb [122]. Studies have also shown that the iodine-induced early increase in thyroid antibody levels is usually transient but is also influenced by genetic and other environmental factors. It appears that adequate iodine intake, not exceeding a urinary iodine concentration of 300 µg, does not increase the risk of AITD according to the literature data, although further investigation is needed [24]. A controlled iodine prophylaxis program is recommended to avoid the adverse effects of iodine deficiency and iodine excess.

#### 4.4.2. Iodine Disbalance During Pregnancy with Autoimmune Thyroid Disease

**Figure 3:**
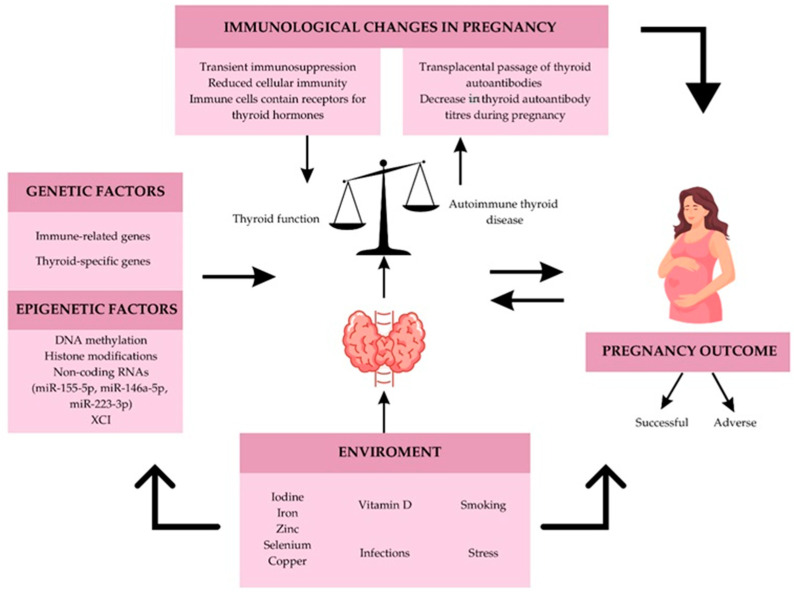
The complex network of factors that influence thyroid function and pregnancy outcome. Both thyroid function and a successful pregnancy are influenced by genetic and epigenetic predispositions as well as environmental factors that, when out of balance, can lead to AITD and an adverse pregnancy outcome. Immunomodulation during pregnancy, which is necessary for a successful pregnancy outcome, also affects thyroid function, especially in the presence of AITD. On the other hand, autoimmunity of the thyroid gland can affect the normal immunological changes and the outcome of pregnancy.

It is well known that increased maternal iodine intake during pregnancy is crucial and that the fetus is dependent on maternal thyroid hormones and maternal iodine reserves in all trimesters of pregnancy. The placenta stores iodine during pregnancy to provide the fetal thyroid gland with a sufficient concentration of iodine for adequate thyroid hormone synthesis and to prevent the negative consequences of iodine deficiency [123]. Adequate maternal iodine intake during pregnancy is required due to increased thyroid hormone production because of elevated levels of β-hCG, circulating estrogens and TBGs. In addition, renal iodine excretion is increased during pregnancy, as is the activity of placental deiodinases [124]. The hormones T4 and T3 are essential for the healthy neurological development of the fetus and are involved in highly sensitive processes such as neuronal migration, myelination, synaptic transmission and plasticity during the fetal and early postnatal period [125,126]. Therefore, it is known that severe iodine deficiency during pregnancy and the neonatal period can have serious adverse effects: an increased risk of pregnancy loss and infant death, neonatal hypothyroidism and neuropsychomotor developmental delay [127]. In addition, a prospective cohort study showed that pregnant women with adequate iodine supplementation had fewer maternal complications such as preeclampsia and placenta previa and a lower rate of fetal distress than pregnant women with severe iodine deficiency [128]. However, there are also studies that have found no association between iodine deficiency and adverse effects such as pregnancy loss, preeclampsia, gestational diabetes, anemia, postpartum hemorrhage, small for gestational age and preterm delivery, among several others [129,130]. Other factors besides iodine deficiency may play a role in pregnancy outcome and further investigation is needed. One of the most important factors to consider is maternal or fetal hypothyroxinemia, which may be exacerbated if iodine deficiency is combined with maternal thyroid autoimmunity during pregnancy. Since hypothyroidism in pregnant women with AITD may worsen during pregnancy, it would be very important to ensure adequate iodine intake to avoid the risk of an unfavorable pregnancy outcome (Figure 3).

As iodine deficiency is preventable and can be avoided by adequate iodine supplementation during and before pregnancy, the importance of developing an effective strategy to avoid iodine imbalance becomes clear. Recent surveillance studies have shown that pregnant women have become moderately iodine deficient over the past decade [131,132]. Similar studies from various countries, including Australia [133], the United Kingdom [106], Spain [112], the Netherlands [113] and the United States [114], have also reported iodine deficiency in pregnant women. This global concern regarding maternal iodine supply has attracted much attention. Recent research in Shanghai also confirms that current iodine intake in pregnant women is inadequate [131].

Although the adverse maternal and fetal consequences of iodine deficiency have been extensively studied and are widely recognized, the effects of iodine excess have not received the same level of attention and warrant further investigation. Iodine is an essential micronutrient that must be obtained through diet, as the body cannot synthesize it on its own. However, excessive iodine intake can also lead to side effects and toxicity [134], especially during pregnancy. There is evidence that subclinical hypothyroidism can develop in pregnant women. An increased incidence of preterm birth has been described, and the neurological development of the fetus may also be impaired [135,136].

Given the significant prevalence of AITD in women of reproductive age and the knowledge that both iodine deficiency and iodine excess can lead to thyroid dysfunction, the careful monitoring of iodine prophylaxis is required to minimize the risk of adverse outcomes during pregnancy. These thyroid dysfunctions necessitate the careful management of iodine levels and monitoring of thyroid function in both the mother and fetus during pregnancy, especially in women with GD. Poor management can lead to notable neonatal morbidity, again emphasizing close monitoring and early intervention [58]. Further research is needed to clarify the impact of iodine status on thyroid autoimmunity, which will greatly contribute to the maintenance of normal pregnancy and the birth of a healthy child.

#### 4.4.3. Selenium and Autoimmune Thyroid Disease: A Scientific Overview

In recent years, selenium, an essential trace element with significant antioxidant properties, has attracted attention for its potential role in AITD. Selenium has the highest concentration of all tissues, emphasizing its essential role in thyroid metabolism. Several selenoproteins have been documented to be expressed in thyroid cells, including the deiodinase isozymes DIO1 and DIO2, which convert T4 to the active hormone T3 [137,138]. These proteins also include glutathione peroxidases (GPX1, GPX3 and GPX4) and thioredoxin reductases (TXNRD1, TXNRD2 and TXNRD3), which regulate thyroid hormone levels and perform oxidoreductase activities. Although DIO3, which inactivates thyroid hormones, is not expressed in thyroid cells, it is expressed in the placenta and plays an important role in fetal development [139]. The thyroid gland is particularly vulnerable to oxidative stress during hormone production, and AITD exacerbates this damage. Selenium is an integral component of various selenoproteins that play a crucial role in redox regulation, thyroid hormone metabolism and immune function. In AITD, immune system attacks exacerbate oxidative damage. The role of selenium in reducing oxidative stress through the function of selenoproteins such as glutathione peroxidase (GPx) and thioredoxin reductase may mitigate tissue damage in the thyroid gland. Given the oxidative stress involved in thyroid inflammation, the antioxidant capacity of selenium, therefore, makes it a potential candidate for therapeutic intervention [140].

A recent large meta-analysis by Huwiler and coworkers [141] has shown that selenium was effective and safe in lowering TSH, TPOAb and malondialdehyde levels. Further studies have shown that selenium supplementation can enhance selenoenzyme activity and help lower inflammatory markers and oxidative stress in patients with HT, which may slow the progression of the autoimmune response and subsequent destruction of thyroid tissue [142]. Since this micronutrient is crucial for the conversion of the inactive hormone T4 to the active form T3 and the conversion is catalyzed by the selenium-dependent enzymes iodothyronine deiodinases, a selenium deficiency can impair this conversion process and contribute to the symptoms of hypothyroidism, even if the thyroid gland produces adequate amounts of T4. Ensuring adequate levels of selenium in the body may also help maintain efficient thyroid hormone metabolism [143].

The influence of selenium on immune function is particularly important in AITD. It can modulate the immune response by influencing both innate and adaptive immunity. Selenium has been shown to regulate the production of pro-inflammatory cytokines and shift the balance of T helper cells, which play a crucial role in autoimmune thyroid destruction. Selenium supplementation has been shown to reduce anti-TPOAbs, associated with the severity of thyroid inflammation and destruction [144,145]. However, mixed results were noted, and this variability in results could be due to differences in baseline selenium status, dosing, duration of supplementation and other patient-specific factors such as genetics and comorbidities. Indeed, long-term studies are needed to determine whether selenium supplementation can prevent the progression of AITD or improve clinical outcomes. In patients with AITD, selenium supplementation at a dose of 200 µg/day is considered safe. However, excessive selenium intake can lead to toxicity (selenosis). Therefore, supplementation should be carefully monitored, especially in regions where selenium levels in food and water are already high [146]. By reducing oxidative stress, modulating immune function and supporting thyroid hormone metabolism, selenium may help to alleviate some aspects of HT, particularly in selenium-deficient individuals. However, until definitive scientific and clinical conclusions are reached, selenium should be considered as an adjunct to conventional treatments under medical supervision [147].

#### 4.4.4. Selenium and Autoimmune Thyroiditis in Pregnancy

Adequate selenium levels are crucial for maintaining appropriate thyroid hormone levels, especially during pregnancy when metabolic demands are increased. The appropriate management of thyroid function during pregnancy is crucial to avoid adverse outcomes. Selenium supplementation has been shown to be a possible adjunct therapy in women with AITD, although further studies are required to confirm its benefit [148]. A recent study showed that selenium levels were low in pregnant women with gestational diabetes compared to controls and selenium supplementation had a positive effect on blood glucose levels [149]. Pregnancy leads to significant changes in immune function, and PPT is a common complication in women with autoimmune thyroiditis. After childbirth, the immune system may be reactivated and attack the thyroid gland, leading to either transient hyperthyroidism or hypothyroidism. Selenium is generally considered safe to take during pregnancy, but dosing is crucial. A randomized controlled trial by Negro et al. examined the effect of selenium supplementation of 200 µg/day in euthyroid pregnant women with AITD and showed that selenium significantly lowered anti-TPO antibody levels during pregnancy and in the postpartum period while reducing the incidence of PPT [150]. These results suggest that selenium may contribute to the stabilization of thyroid function during pregnancy. Animal studies and observational studies have shown that selenium supplementation may support normal fetal development by ensuring adequate thyroid hormone levels [151]. Some studies suggest that selenium deficiency may be associated with neurodevelopmental delays in children. However, further research is needed to establish a direct link between maternal selenium intake and improved cognitive performance in offspring [152,153].

Although a positive role of selenium supplementation on thyroid function during pregnancy is suspected, especially in pregnant women with overt or subclinical hypothyroidism, most guidelines do not suggest routine selenium supplementation in pregnancy due to an overall lack of studies [59]. A 2017 review by Ventura et al. showed that selenium may contribute to thyroid health. However, they found that most studies measured thyroid antibodies but not selenium levels themselves. Since selenium has a narrow therapeutic index, supplementation could easily lead to toxicity [154]. Selenium supplementation may prevent complications such as preeclampsia, miscarriage and PPT, although this is not yet well established. Selenium plays an important role in the synthesis of thyroid hormones and antioxidant protection, but its supplementation regarding pregnancy complications is still under investigation [155].

In areas where selenium is deficient, selenium supplementation has also been considered to improve pregnancy outcomes, for example, by reducing preterm births, small-for-gestational-age babies and pregnancy-induced hypertension. Selenium supplementation, especially in the form of selenomethionine, has been shown to significantly lower levels of antithyroid anti-TPOAbs in euthyroid women with AITD throughout pregnancy. It may prevent the progression of hypothyroidism and reduce the inflammatory response during pregnancy, leading to better maternal and fetal outcomes. Selenium supplementation, while promising, is only recommended in some cases. Both the American and European Thyroid Associations urge further research to determine the optimal dosage and safety of routine selenium use during pregnancy. The benefits of selenium are more pronounced in selenium-deficient regions; therefore, geographic considerations are an important variable when making supplementation recommendations [69].

#### 4.4.5. Autoimmune Thyroid Disease, Pregnancy and Iron—Causal Connections and Implications

Recent evidence emphasizes the role of iron in thyroid hormone synthesis and immune function, making iron status a very important factor in the management of AITD in pregnancy [156]. Iron plays a crucial role in both thyroid function and immune system regulation, making it particularly important for pregnant women with AITD. Iron is a co-factor for TPO, which is essential to produce thyroid hormones. If the iron supply is insufficient, the activity of TPO is impaired, which leads to the reduced synthesis of thyroid hormones [157]. Iron deficiency can impair the production of thyroid hormones and, thereby, exacerbate hypothyroidism, especially in women with AITD, which can lead to complications such as fatigue, anemia or the worsening of thyroid dysfunction [158]. Iron plays an important role in regulating the immune system and helps to maintain the balance between pro-inflammatory and anti-inflammatory responses. Iron deficiency can impair immune function and make the body more susceptible to exaggerated inflammatory responses, potentially exacerbating thyroid autoimmunity, which can lead to increased thyroid damage [159].

Low iron and ferritin levels during pregnancy can lead to anemia, which, in combination with thyroid dysfunction, can increase the risk of miscarriage, preeclampsia and premature birth. Women with AITD already have a higher risk of these complications, so iron deficiency further increases the risks [160]. Adequate thyroid function is critical for fetal neurologic development, especially in the first trimester, and iron deficiency predicts poor maternal thyroid status during pregnancy [161]. Since the fetus is dependent on maternal thyroid hormones, iron deficiency leading to hypothyroidism may impair fetal brain development and increase the risk of cognitive impairment and developmental delay [162]. For pregnant women with AITD, the monitoring and treatment of iron levels is crucial. Therefore, the appropriate management of iron levels is critical for optimizing maternal and fetal health. Iron supplementation may be necessary to prevent the exacerbation of thyroid dysfunction as it helps to stabilize TPO activity, improve thyroid hormone production and reduce the risk of complications [159].

#### 4.4.6. Vitamin D and Thyroid Dysfunction

A growing body of research suggests that vitamin D, which is also a secosteroid hormone, may play a role in thyroid health and the development of thyroid disease [163]. Vitamin D receptors (VDR) are found in many tissues, including immune cells, suggesting that they play a critical role in regulating the immune response. The active form of vitamin D, 1,25-dihydroxyvitamin D (calcitriol), exerts immunoregulatory effects, particularly in autoimmune diseases. Scientific evidence confirms that insufficient vitamin D levels can lead to immune dysregulation, possibly contributing to autoimmune thyroid diseases, although conflicting results have been found [164]. Nevertheless, numerous studies have shown that patients with HT tend to have lower vitamin D levels compared to healthy controls. The presence of TPOAb and lower vitamin D levels are often associated with higher antibody titers, indicating a more active autoimmune process [165]. Similarly, hyperthyroidism due to GD has been associated with lower vitamin D levels. Studies suggest that vitamin D deficiency may exacerbate disease severity, although the scientific evidence is less consistent compared to hypothyroidism [166,167].

Several biological mechanisms have been proposed that could explain the link between vitamin D deficiency and thyroid dysfunction, of which the immuno-modulatory effect of vitamin D seems to be the most important [168]. Vitamin D is known to regulate both innate and adaptive immune responses and can modulate the proliferation of T cells and the differentiation of T-regs, which are crucial for the maintenance of immune tolerance [169]. Vitamin D deficiency may impair these mechanisms and, thus, contribute to the development and/or progression of AITD and other autoimmune diseases [170,171]. In addition, VDRs are expressed in thyroid cells, suggesting that vitamin D may influence thyroid function. Recent findings show an association between VDR gene polymorphism, vitamin D status and AITD [172]. Vitamin D is also known for its anti-inflammatory properties, and its deficiency may lead to increased inflammation and oxidative stress in thyroid tissues. Chronic inflammation is a key component of AITD, and vitamin D supplementation has been suggested as a potential means of reducing this inflammation, although clinical trials examining the effects of vitamin D supplementation on thyroid function are still limited [173].

Numerous epidemiological studies have shown a link between low vitamin D levels and thyroid disease [174]. However, the question of whether vitamin D deficiency is a cause or a consequence of thyroid dysfunction remains a topic of ongoing research, as the results are not yet conclusive. Indeed, further research is needed to determine whether vitamin D supplementation can serve as a therapeutic or preventive measure for thyroid dysfunction.

#### 4.4.7. Smoking and Autoimmune Thyroid Disease

Cigarette smoking is considered one of the environmental factors affecting thyroid function and appears to have different effects on the development of autoimmune thyroid disease. Data on the effects of cigarette smoking on thyroid function and the triggering of thyroid autoimmunity are varied and conflicting. It has been suggested that the chemical components of tobacco may affect hormone production, hormone transport and secretion [175]. Smoking has been reported to increase the size of the thyroid gland and the development of non-toxic goiter [176]. There are studies showing that smokers have higher thyroid hormone levels and lower TPOAb levels than non-smokers [177], but other studies have not confirmed these results [178,179]. The results of most studies relied on questionnaires to assess smoking status, which is associated with limitations such as information bias and subjectivity [175]. Several studies investigated serum cotinine as a measure of nicotine exposure to more objectively determine smoking status [180,181]. Soldin et al. reported decreased TSH and T4 levels in active smokers [182], while Kim et al. found that cigarette smoking was associated with decreased TSH levels in men and women and increased TPOAb levels in male subjects [175]. In addition, they reported that urinary cotinine levels were negatively associated with TSH levels after controlling for age, height, weight, health behaviors and urinary iodine levels. The authors suggest that smoking may have a stimulating effect on the thyroid gland by increasing serum TBG and T3 concentrations and lowering serum TSH levels [175]. Cigarette smoking may impair iodide transport and iodide organization. One hypothetical explanation is that thiocyanate, a metabolite of cigarette smoke, inhibits the sodium iodide symporter and, thus, impairs iodine uptake and thyroid function, especially in iodine-deficient women. This could lead to the development of autoimmune thyroid dysfunction and increased production of thyroid hormones, which, in turn, lowers TSH levels [183,184]. This was supported by the research conducted by Shields et al., which showed that smoking lowers thyroid antibody levels, possibly due to the inhibitory effect of thiocyanate, on the sodium iodide symporter, which affects iodine uptake in the thyroid gland. It was also found that smokers were less likely to test positive for TPOAbs, again supporting the hypothesis of immune modulation by smoking that suppresses the autoimmune responses of the immune system, thus explaining the lower risk of hypothyroidism in active smokers. The study also found that this protective effect wears off after quitting smoking and that people return to their baseline risk of hypothyroidism a few years after quitting [185].

To date, cigarette smoking has been shown to be associated with a twofold increased risk of GD and a higher likelihood of relapse in GD or GO [16,186]. The mechanism is not yet fully understood, but smoking is thought to increase the risk by increasing the formation of reactive oxygen species and decreasing the production of antioxidants [16]. However, the effects of smoking in HT are not so clear and mixed results have been reported. There is evidence that cigarette smoking is associated with a lower prevalence of TPOAb [176,187,188]. On the other hand, there are studies that find no significant correlation between smoking and TPOAbs [189]. Although the data are contradictory, smoking appears to lower the overall risk of TPOAb, TgAbs and autoimmune hypothyroidism by a factor of about 40%. It has been suggested that nicotine alters immune responses by activating nicotinic receptors on immune cells, shifting the autoimmune profile away from the Th1 and Th17 pathways [190]. Smoking can impair cell-mediated immunity and inhibit the activity of natural killer cells and reduce the number of cytotoxic CD8+ T cells [191] which could reduce the risk of the thyroid autoimmunity [176]. In addition to autoimmunity and iodine status, the effect of smoking on thyroid function might depend on several factors, such as age, the duration and intensity of smoking, physical health and comorbidities and other environmental factors [192].

#### 4.4.8. Smoking and Thyroid Function During Pregnancy

Smoking during pregnancy can have harmful effects on the development of the fetus. It is associated with an increased risk of miscarriage, growth failure and malformations, e.g., orofacial cleft, congenital heart defects or neural tube defects [193,194,195,196]. Quelhas et al. reported that active tobacco use during pregnancy was associated with significantly higher rates of small for gestational age, shorter length and smaller head circumference at birth [193]. One of the suggested mechanisms is oxidative stress in the placenta caused by tobacco, which can impair the transport of oxygen and nutrients to the growing fetus [197]. Yuan et al. showed, in a Mendelian randomization investigation, a positive association between smoking initiation and increased risk of pregnancy loss [198]. A long-term consequence was also found to be impaired intellectual development [199]. However, data on the effects of smoking on thyroid function during pregnancy are limited and contradictory. Smoking during pregnancy has been shown to increase the risk of subsequent hyperthyroidism, while paradoxically, it also has a protective effect against hypothyroidism [59]. Andersen et al. showed lower TSH, higher T3 and lower or unchanged T4 in pregnant women who smoked [200]. One of the possible explanations is that smoking affects the activity of deiodinase, especially D2, during pregnancy and the delicate balance of the sympathetic nervous system [124]. Andersen et al. also reported that women who quit smoking during pregnancy had higher TSH, lower T4 and higher T3 [200]. Previous research suggests that smoking lowers the risk of positive thyroid autoantibody concentrations, which may be one reason for the protective effects of smoking on hypothyroidism in this population. Shields et al. investigated thyroid function in a cross-sectional study of two independent cohorts of pregnant women without a history of thyroid disease or with overt biochemical thyroid dysfunction [185]. They reported that pregnant women who smoked had lower TSH and higher FT3 levels than non-smoking pregnant women, while FT4 levels were similar in both groups, as was the prevalence of TPOAbs. TSH levels were also lower in the cord blood of babies born to mothers who had smoked during pregnancy [185]. A meta-analysis showed that smoking was associated with an increased risk of both HT and GD. In the population at risk for AITD, an inverse association was found between smoking and the presence of TPOAb, a finding that was later confirmed in other population-based studies [16]. Curiously, smoking during pregnancy was associated with a lower risk of developing thyroiditis, but increased the incidence of PPT [185].

Despite this protection against hypothyroidism, smoking during pregnancy carries many other risks, including an increased risk of low birth weight, preterm birth and infant mortality. Healthcare providers should, therefore, watch for symptoms of hyperthyroidism in women who smoke, especially in the first two years after giving birth. Although smoking during pregnancy has decreased, a large proportion of pregnant women still smoke, which means that public health efforts need to continue.

Clearly, further research is needed to investigate the effects of maternal thyroid autoimmunity and smoking on fetal development and adverse pregnancy outcomes and to identify the complex underlying mechanisms.

#### 4.4.9. Infections and Autoimmune Thyroid Disease

The immunological basis of AITD has both an innate and an adaptive component. The innate mechanisms of immune responses are primarily cell-mediated and represent a rapid defense against pathogens, whereas the adaptive mechanisms are antigen-specific and elicit antibodies against specific targets. Autoimmune thyrocyte destruction in HT is a cell-mediated phenomenon represented by lymphocytic infiltration into thyroid follicles. Recent evidence suggests that stromal cells in the thyroid gland of HT patients may drive the recruitment of inflammatory cells into organized lymphoid structures called tertiary lymphoid organs. The resulting tissue destruction leads to exposure to thyroid antigens, triggering the production of autoantibodies. In contrast, GD shows less aggressive lymphocytic infiltration and a predominant humoral immune response, although these processes are interdependent [83].

While adaptive immunity in AITD is well documented, recent attention has focused on the innate immune cells involved, such as neutrophils, NK cells, NKT cells, monocytes, macrophages and dendritic cells [83,201]. The activation of innate immune pathways usually leads to the release of cytokines, infiltration of lymphocytes and tissue destruction. Although innate immunity is classically considered to be non-specific, some degree of specificity has been established [202].

NK cells are components of innate immunity and work together with T lymphocytes as major effectors of cell-mediated immunity. NK cells make up 10–15% of lymphocytes in peripheral blood and have been categorized into two subsets based on the cell surface density of the neuronal cell adhesion molecule (N-CAM), also known as CD56: one subset comprises cells often referred to as CD56^dim^ cells, which are highly cytotoxic and account for 90% of NK cells, and the remaining fraction consists of CD56^bright^ cells, which are immunoregulatory cells and produce cytokines [203,204]. Due to their cytotoxic nature, they can destroy virus-infected and malignant cells without prior sensitization, a unique property compared to T lymphocytes. NK cells are activated by the proinflammatory cytokines IL-2, IFN-γ, IFN-β and IL-12 and may take on regulatory functions via their interactions with T cells and dendritic cells, thereby modulating both innate and acquired immunity [203].

In viral infections, NK cells play the most important role due to their ability to rapidly eliminate virus-infected cells. The activity of NK cells is controlled by a delicate balance of activating and inhibitory receptors on their surface that control cytotoxic responses [205]. NK cells kill target cells via two main pathways: a direct cytotoxic release of granules and binding to “death receptors” such as Fas/FasL on the target cells. The activated NK cells produce cytokines such as IFN-γ, TNF-α and TNF-β, which help in immune regulation and apoptosis. NK cells are also involved in autoimmune diseases such as multiple sclerosis and Crohn’s disease, where viral antigens interfere with NK cell function. Chronic viral infection has been shown to downregulate NK cytotoxicity, promote viral persistence and contribute to autoimmunity through its effects on B- and T-cell responses.

During pregnancy, there is a functional change in NK cells. In a normal pregnancy, the number and activity of NK cells increase in the first trimester and then decrease in the later stages. However, high NK cell activity has been associated with miscarriage and recurrent pregnancy loss, suggesting a role in immune regulation at the feto-maternal interface [83]. The exacerbation of autoimmune thyroid disorders of AITD, such as HT and GD after delivery, has also been associated with increased NK cell activity [205]. These findings emphasize that NK cells play a Janus-like role both in the protection against viral infections and the development of autoimmune diseases and pregnancy complications. In summary, NK cells play a crucial role in viral immunity and the control of autoimmunity, and functional changes in diseases such as AITD, viral infections and pregnancy suggest that they are involved in the immune response [206].

#### 4.4.10. Stress and Autoimmune Thyroid Disease

There is growing evidence that stressful life events are among the environmental fac-tors that may contribute to the development of autoimmunity in genetically predisposed individuals [207]. Stress is known to lead to the excessive secretion of glucocorticoids and catechol-amines through activation of the hypothalamic–pituitary–adrenal axis and sympathoadrenal system, which can disrupt immune homeostasis and cause the Th1/Th2 imbalance associated with autoimmunity and the pathogenesis of AITD [208]. Elevated glucocorticoid levels affect the cytokine network by inhibiting the synthesis of IL-1, IL-2, TNF and IFN and stimulating the production of IL-4, IL-10 and IL-13, thereby shifting the immune profile towards a humoral response, which is mainly involved in the etiopathogenesis of GD [209]. There is also evidence that elevated glucocorticoid levels can downregulate antioxidant enzymes [210]. There are studies that establish a link between stress and the development of GD [115]. However, the results are not yet clear. Stressful life events have been reported to favor the onset and recurrence of GD in patients who have been observed for at least 5 years after the exclusion of thyrostatic therapy [211]. On the other hand, there was a prospective 5-year follow-up study that showed no causal relationship between stress and GD [208,212].

The influence of stress on the development of HT is also far from clear, although there are accumulating data suggesting that the pathogenesis of HT is more complex and involves both cellular and humoral immune mechanisms [213]. The authors also suggested that acute stress could lead to the progression of HT, while chronic stress induces a Th2 immune response and increased levels of TPOAb and Tg autoantibodies [209]. Interestingly, Vaivode et al. investigated the relationship between the number and impact of stressful life events in AITD patients and the Th1/Th2/Th17 immune response and found no significant differences in the number of stressful life events between patients with HT, patients with GD and controls. There was a positive correlation between the number of major life events and life events with negative effects on TPO and Tg antibody levels [208]. The exact role of TPOAbs in the pathogenesis of HT is not clear, but they appear to be associated with oxidative stress, as higher oxidative parameters have been found in euthyroid, untreated HT patients [214]. Markomanolaki et al. reported that after an 8-week stress management intervention, patients showed a statistically significant decrease in TgAb titers and stress, depression and anxiety scores compared to the control group [213]. The study by Corso et al. provides the first evidence that stressful events such as emotional neglect and abuse are potential risk factors for the development of AITD [215]. In a pilot study investigating the relationship between thyroid function and perceived stress in newly diagnosed hypothyroid women of reproductive age, a significant difference was found between clinical and subclinical hypothyroid women in terms of the mean score of the Perceived Stress Scale (PSS), which measures the individual perception of people exposed to stressful situations. A significant positive correlation was found between PSS scores and TSH levels [207]. A recent cross-sectional study also showed a positive but non-significant correlation between PSS and TSH in women of reproductive age with normal thyroid function and with SH [216].

#### 4.4.11. Stress and Thyroid Function During Pregnancy

Data from the literature suggest that stress during pregnancy increases the risk of pregnancy complications and unfavorable outcomes such as low birth weight, preterm birth [217] and impaired fetal neurodevelopment [218]. Maternal stress has also been associated with an increased risk of pregnancy loss [219], gestational diabetes [220], birth complications [221,222], hypertension and pre-eclampsia [223]. Stressful events during pregnancy can cause changes in glucocorticoid levels through activation of the hypothalamic–pituitary–adrenal axis. It has been found that both decreased and increased concentrations of glucocorticoids can affect fetal neurodevelopment [224]. Glucocorticoids are involved in structural and neurochemical processes that are important for brain maturation [225].

The literature data on the effects of stress on pregnancy with AITD are insufficient, but it has been shown that activation of the hypothalamic–pituitary–adrenal axis and increased glucocorticoids affect thyroid hormone metabolism during pregnancy by lowering TSH levels and inhibiting the peripheral conversion of T4 to the active T3 form [226]. It has also been described that glucocorticoids can alter iodine metabolism, thyrocyte secretory activity and the differentiation of deiodinase activity. Thyroid hormones are essential for normal fetal development, especially for brain maturation and neurophysiological processes such as neuron and glial cell differentiation and synaptogenesis [227]. T3 plays the main role in fetal neurodevelopment, and T3 receptors are already present in the brain in early pregnancy. Most of the fetal T3 in the central nervous system is locally converted from maternal T4, which is actively transported into the fetal central nervous system [227]. In pregnant women with HT and reduced thyroid reserve due to an autoimmune process, stress-related events could further jeopardize transplacental delivery and the supply of T4 to the growing fetus. Therefore, stress-related abnormalities in thyroid hormone and glucocorticoid levels during pregnancy, especially in autoimmune hypothyroidism, could have short- and long-term consequences for the offspring. We cannot rule out stressful events during pregnancy, but maintaining maternal TSH and thyroid hormone levels within a strict normal range contributes to a successful pregnancy and birth, as well as safe and normal fetal development.

## 5. Conclusions

During pregnancy, maintaining normal thyroid function is crucial for both the mother and the developing fetus. Since maternal and fetal thyroid function are closely interconnected, any changes in the mother’s thyroid health can influence the course and outcome of the pregnancy, as well as the condition of the fetus. AITD is relatively common in pregnant women, and pregnancy itself can exacerbate AITD due to various hormonal changes. Often, AITD remains undiagnosed and untreated, particularly in its subclinical form. Therefore, both preventing the onset of AITD and managing existing cases during pregnancy are of utmost importance. To effectively address AITD in pregnancy, it is essential to understand the factors that contribute to its development and take steps to mitigate them. Current knowledge suggests that AITD develops in genetically predisposed individuals, influenced by environmental factors and mediated by epigenetic mechanisms. The genetic background plays a role in immune regulation and may predispose the thyroid gland to autoimmune responses triggered by environmental factors. While modifiable environmental factors present a promising target for the prevention and treatment of AITD, there is still a lack of established protocols on how to intervene effectively. At present, ensuring adequate iodine intake remains a key focus, with iodized salt being the primary method of implementation. Research has also linked insufficient selenium and vitamin D levels to thyroid autoimmunity during pregnancy, but routine supplementation with these nutrients is not yet recommended. Additionally, avoiding smoking and managing stress have been shown to have beneficial effects on thyroid health. One promising approach is systematic screening for AITD during pregnancy, which could significantly help in preventing adverse pregnancy outcomes and developmental disorders in the child. However, due to the substantial financial resources and health system involvement required, this approach has not yet been widely implemented.

## Data Availability

No new data were generated with this research.
